# PEGylation of Deferoxamine for Improving the Stability, Cytotoxicity, and Iron-Overload in an Experimental Stroke Model in Rats

**DOI:** 10.3389/fbioe.2020.592294

**Published:** 2020-09-25

**Authors:** Jiake Xu, Tong Sun, Rui Zhong, Chao You, Meng Tian

**Affiliations:** ^1^Neurosurgery Research Laboratory, National Clinical Research Center for Geriatrics, West China Hospital, Sichuan University, Chengdu, China; ^2^Department of Neurosurgery, West China Hospital, Sichuan University, Chengdu, China; ^3^Peking Union Medical College, Institute of Blood Transfusion, Chinese Academy of Medical Sciences, Chengdu, China; ^4^West China Brain Research Centre, West China Hospital, Sichuan University, Chengdu, China

**Keywords:** Poly(ethylene glycol), deferoxamine, stability, cytotoxicity, hemocompatibility, iron overload, stroke

## Abstract

Deferoxamine (DFO) is a widely used drug for the treatment of iron-overload-related diseases in the clinic. However, its inherent shortcomings, such as a short plasma half-life and cytotoxicity, need to be addressed to widen its clinical utility. In this study, PEGylated DFO was first synthesized, and its chemical structure was characterized, and then *in vitro* and *in vivo* studies were performed. The metabolism assay showed that the stability of the PEGylated DFO was significantly improved, with a half-life 20 times greater than DFO. Furthermore, the PEGylated DFO exhibited significantly lower cytotoxicity compared with DFO. Additionally, the hemocompatibility assay showed that the PEGylated DFO had no significant effect on the coagulation system, red blood cells, complement, and platelets. *In vivo* studies indicated that PEGylated DFO was capable of reducing the iron accumulation, degeneration of neurons, and promotion of functional recovery. Taken together, PEGylated DFO improved stability, cytotoxicity, and iron-overload in an experimental stroke model in rats, making it a promising therapy for treating iron-overload conditions in the clinic.

## Introduction

Iron-overload is a serious problem that commonly presents in the clinic and is associated with many diseases such as hereditary hemochromatosis, thalassemia, Alzheimer’s disease, Parkinson’s disease, and stroke. This is because mammals, such as humans, cannot secrete excess iron in a controlled manner (Camaschella et al., 2020). To treat these conditions, iron chelation therapy has long been the gold standard for treatment, and one of the commonly used drugs is deferoxamine (DFO) (Holden and Nair, 2019). However, the clinical use of DFO has been limited by its inherent shortcomings. One of these shortcomings is the rapid metabolism of DFO by the globulin in the blood, resulting in a short plasma half-life (30 min). Therefore, long-term and frequent administration of DFO was required, which in turn resulted in poor compliance for patients. Furthermore, DFO toxicity is dose- and time-dependent, and results in complications such as cardiomyopathy, growth retardation, and endocrine dysfunction (Porter, 1997; Baath et al., 2008; Tian et al., 2016a; Guo S. et al., 2018).

The development of a novel drug delivery approach may serve as an effective strategy to overcome these inherent shortcomings and protect the drug from its rapid metabolism and accumulation at the lesion site. Thus, this approach may not only improve the stability and prolong the half-life of the drug but also permit the drug to be administered at lower frequencies and dosages, relieving the toxicity (Haag and Kratz, 2006; Tibbitt et al., 2016; Xiao et al., 2018; Feng et al., 2019). Previous reports indicated that the half-life and toxicity of DFO were improved when delivery carriers were used (Rossi et al., 2009; Tian et al., 2016a; Sun et al., 2020). Hallaway et al. (1989) reported that the plasma half-lives increased more than 10-fold for dextran-DFO and hydroxyethyl starch-DFO. Similarly, when DFO was conjugated to hyperbranched polyglycerol, the plasma half-life was also significantly increased. Additionally, the toxicity (assayed *in vitro* by hemocompatibility assays and *in vivo* in mice) showed that there was no significant effect on blood components, including red blood cells, coagulation system, complement, and platelets. Furthermore, apparent toxicity was not detected when measured using changes in body weight, serum lactate dehydrogenase levels, necropsy analysis, and histopathological examination of organs (Imran ul-haq et al., 2013).

The ideal delivery carrier should be degraded and cleared from the body to avoid its accumulation in tissues. A recent study reported that an alginate carrier induced an oxidative response when conjugated to DFO. These results also showed that the conjugates had a half-life more than 10 times longer and reduced cytotoxicity when compared with DFO. In contrast, the coagulation system was significantly affected, that is, the coagulation time, particularly activated partial thromboplastin time (APTT) and thrombin time (TT), were significantly prolonged in a dose-dependent manner. Fibrinogen was dramatically decreased, suggesting that the conjugates could dominantly inhibit the intrinsic pathways in the process of coagulation (Sun et al., 2020). Therefore, this significant side effect and its impact on patient safety should also be considered when designing the delivery carrier to be utilized for DFO.

**FIGURE 1:**
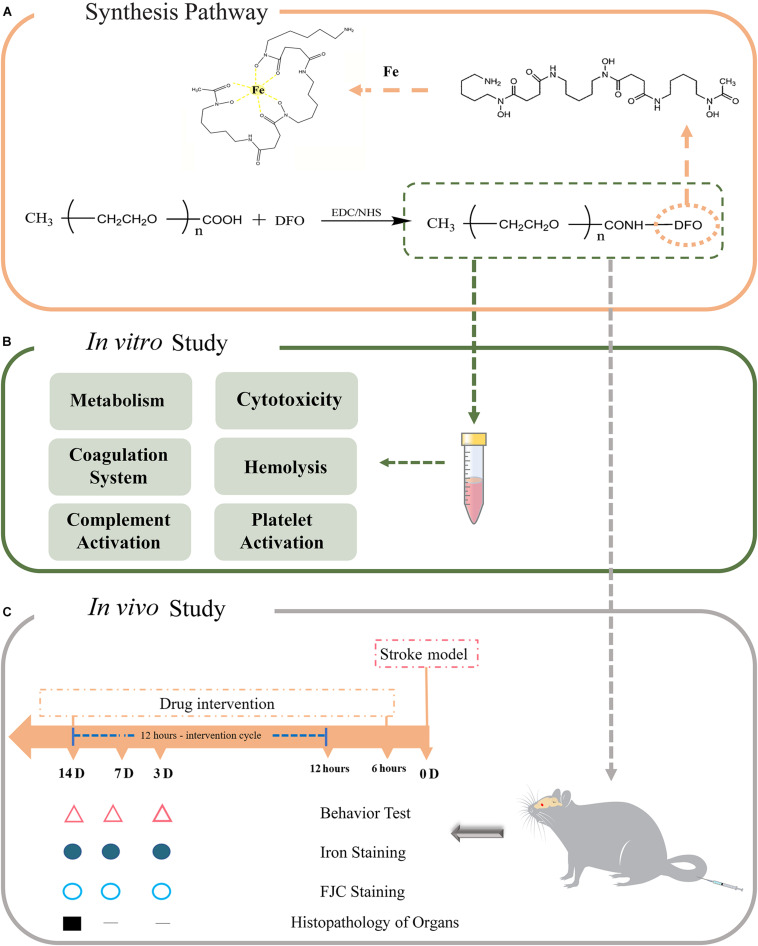
**(A)** The synthesis pathway of PEGylated DFO. **(B)** The *in vitro* study of PEGylated DFO. **(C)** The *in vivo* study of PEGylated DFO in a stroke model.

Poly(ethylene glycol) (PEG) is an FDA approved biocompatible polymer that is widely used as a delivery carrier for the modification of proteins, peptides, or other drugs by chemical linking. This process is defined as PEGylation, and the final product is non-toxic, non-immunogenic, non-antigenic, and highly soluble in water. More importantly, PEGylation prolongs the half-life and decreases the toxicity of drugs, and thus it seems that PEGylation is appropriate for DFO modification (Alconcel et al., 2011; Sun et al., 2015; D’souza and Shegokar, 2016; Suk et al., 2016). In this study, we synthesized PEGylated DFO to increase its half-life and decrease the toxicity of DFO (without side effects and patient safety concerns). To address this hypothesis, the PEGylated DFO was first synthesized by EDC coupling chemistry, and then *in vitro* assays, including metabolism, cytotoxicity, and hemocompatibility, were performed (Figure 1). Finally, the extent of *in vivo* iron elimination and its therapeutic effects were evaluated in an experimental stroke model in rats.

## Materials and Methods

### Synthesis of PEGylated DFO

Synthesis of PEGylated DFO was performed using EDC coupling chemistry. Briefly, 1g of carboxylated mPEG (molecular weight: 2 and 5 kDa) was dissolved in 100 ml HEPES buffer (pH 6.8), and then DFO, EDAC, and NHS were added as solids to the reaction with a molar ratio of -COOH/DFO/EDAC/NHS 1:2:2:1. After a 24 h reaction time, the resulting solution was dialyzed (500 Da molecular weight cut-off) against super pure water. The final solution was clarified using centrifugation, and the supernatant was sterilized with a Millipore filter (0.2 μm) and then lyophilized. The resulting products were stored at -20°C. The products synthesized from carboxylated mPEG with molecular weight 2 and 5 kDa are referred to as mPEG_2__k_-DFO and mPEG_5__k_-DFO, respectively.

### Characterization of PEGylated DFO

The products were ground with KBr powder and compressed into pellets for FTIR spectroscopy analysis. The spectra of the samples were recorded as transmittance using a Nicolet 670 FTIR Spectrometer (Thermo Fisher Scientific, Waltham, MA, United States). ^1^H NMR analyses were recorded on a Bruker AV II-400 MHz spectrometer at 298 K. Each sample (10 mg) was dissolved in 1 ml D_2_O before spectroscopic determination.

### Chelation Ability of PEGylated DFO

The chelation ability of PEGylated DFO and amounts of DFO in the products were determined using UV-Vis spectroscopy (Hallaway et al., 1989). Briefly, each product was dissolved and diluted with ferrous sulfate solution and left to stand overnight at room temperature. The DFO content was calculated according to the absorbance at 429 nm using a standard curve. The amount of DFO was described as the number of DFO molecules coupled per mPEG chain.

### Stability of the PEGylated DFO

The stability of the PEGylated DFO was investigated by metabolism experiments. Plasma for this experiment was obtained from 8-week-old male, Sprague Dawley rats. The protocol was approved by the Animal Ethical Committee of Sichuan University, and Chinese national guidelines for the care and use of laboratory animals were applied. The metabolism experiments were carried out according to our previous reports (Tian et al., 2016a, b).

### Cytotoxicity of the PEGylated DFO

The cytotoxicity of the PEGylated DFO was assayed using the MTT method described in our previous reports (Sun et al., 2020). Briefly, human umbilical vein endothelial cells (HUVECs) were harvested, and after three passages, they were seeded in 96-well plates at a density of 3 × 10^3^ cells/well. After 24 h, the culture medium was replaced with PEGylated DFO or DFO solutions containing equivalent concentrations of DFO (0.5, 0.2, 0.1, 0.05, 0.01, and 0.005 mM) supplemented with 10% FBS, 100 μg/ml penicillin and 100 μg/ml streptomycin. Two days later, the culture medium was changed with fresh medium, and 20 μl of MTT solution (5 mg/ml) was added to each well and incubated at 37°C for 4 h. Next, 150 μl of DMSO was added to the wells and shaken for 10 min. The optical density of each well was determined using a microplate reader at a wavelength of 490 nm. Normalized viability (%) was expressed by the ratio of the optical density of the conjugate (or DFO) to the control.

### Hemocompatibility of the PEGylated DFO

This experiment was approved by the Ethical Committee of the Institute of Blood Transfusion, Chinese Academy of Medical Sciences and Peking Union Medical College. The blood samples were collected from three healthy donors at Chengdu Blood Center after obtaining written informed consent for the use of their blood samples.

The hemocompatibility of the PEGylated DFO was assayed by studying its effects on various blood components. These components include the coagulation system [activated partial thromboplastin time (APTT), prothrombin time (PT), thrombin time (TT), and the concentration of fibrinogen (Fib)] red blood cells (hemolysis), platelet (activation), and complement (C3a, and C5a activation). We referred to the experimental protocols from our previous reports (Guo X. et al., 2018; Sun et al., 2019).

### *In vivo* Study

#### Stroke Model

All animal experiments were processed under the protocol approved by the Animal Ethical Committee of Sichuan University. Fifty-four adult male Sprague-Dawley rats for this study (6–7 weeks old and 200–220 g, Dashuo Laboratory Animal Co., Ltd., Chengdu, China) were housed with the light/dark (12/12 h) cycle conditions and free access to food and water.

The autologous blood model was chosen in this study (Okauchi et al., 2009; Auriat et al., 2012). The rats were anesthetized with 10% chloral hydrate intraperitoneally (0.4 mL/100 g, 10%) and secured in the Stereotactic Instruments (Stereotactic, Single Manipulate; 18 Degree Ear Bars; RWD Life Science, Shenzhen, China). We used stereotactic coordinates to localize the basal ganglia: 0.2 mm anterior, 5.5 mm ventral, and 3.5 mm lateral to the bregma. A burr hole of 1 mm diameter was drilled on the skull at 0.2 mm anterior and 3.5 mm lateral (right) of bregma with a micro drill (RWD Life Science).

Autologous whole blood from the tail artery was injected from the hole (5.5 mm depth below the surface of the skull) using a double-injection method (Shrestha et al., 2018). The needle was pulled out slowly (1 mm/min) after remaining in position for 10 min, and the skull hole was sealed with bone wax. Finally, the skin incision was sutured, and then the animal was placed in a heating blanket (ALC-HTP Constant Temperature System for Animals; Alcott Biological Science and Technology Co., Shanghai, China) until it woke up.

#### Experimental Groups

The rats were randomly assigned to three groups (*n* = 54, and 6 mice per time point): saline (normal saline)-treated, DFO-treated, and mPEG_5__k_-DFO-treated (which is similar to mPEG_2__k_-DFO in cytotoxicity and blood compatibility, but with longer biological half-life). The intravenous DFO and mPEG_5__k_-DFO injections (100 mg/kg mixed with 0.5 mL of saline, prepared fresh at the time of use) were started 6 h after creating the experimental stroke model and repeated every 12 h for 14 days. The saline group received 0.5 mL saline injection at each time point for 14 days.

#### Histological Examination

Paraffin embedding was performed after cardiac perfusion on days 3, 7, and 14 respectively, and cut into 3 mm-thick coronal sections with a microtome (Leica RM2235, Germany). Iron staining was used to observe iron deposition around the lesion. After deparaffinization, the sections were incubated in iron stain [5% potassium ferrocyanide (Kemiou Chemical Reagent Co., Tianjin, China)/5% hydrochloric acid] for 30 min. This was followed by rinsing in water, counterstaining in nuclear fast red (Leagene Biotechnology, Peking, China) for 15 min and then rinsing again in water. Next, the sections were dehydrated in alcohol, and the sections were mounted with mounting medium. The staining sections were observed with a light microscope (BX41, Olympus, Tokyo, Japan).

Fluoro-Jade C (FJC) staining was used to quantify neuronal degeneration. After deparaffinization, the sections were immersed in 0.06% potassium permanganate solution for 10 min and then transferred into a 0.1% acetic acid solution containing 0.0001% FJC (catalog no. AG325-30MG, Merck Millipore, Burlington, MA, United States) for 30 min. Next, the sections were rinsed in water and dried in an air oven (50°C), then they were coverslipped with DPX mounting medium (Sigma-Aldrich). The staining sections were observed through a fluorescence microscope (AX10 imager A2/AX10 cam HRC; Carl Zeiss, Germany).

Three sections per rat and three randomly selected locations per section around the lesion area were used for the quantitative analysis of iron- or FJC-positive cell staining. The results were measured by a blind observer using the open-source software ImageJ/Fiji (US National Institutes of Health). The number of iron- or FJC-positive cells were presented as the average of positive cells per square millimeter.

For the Histopathology of organs, the heart, liver, spleen, lung, and kidney of normal, Saline, DFO, and MPEG_5__k_-DFO groups were removed at necropsy and No abnormalities of the organs were observed during the necropsy. After fixed in 10% buffered formalin, Multiple pieces of tissue were removed from each organ for paraffin embedding and treated by hematoxylin and eosin (H&E) to observe the change of morphosis (light microscope, BX41, Olympus, Tokyo, Japan).

#### Neurological Behavior

To assess the neurobehavioral recovery of the rat, seven neurological deficit tests were evaluated by the blinded investigator on days 3, 7, and 14 after creating the experimental stroke model. The neurological deficit tests (score between 0 and 4 for each item, maximum score = 28) include body symmetry, gait, climbing, circling behavior, front limb symmetry, compulsory circling, and whisker (Hazel, 1998).

### Statistical Analysis

We used analysis of variance (ANOVA) and paired *t*-tests for the statistical analysis. A probability *P* < 0.05 was considered to have a significant difference and was calculated using SPSS 19.0.

## Results and Discussion

### Synthesis and Characterization of PEGylated DFO

**FIGURE 2:**
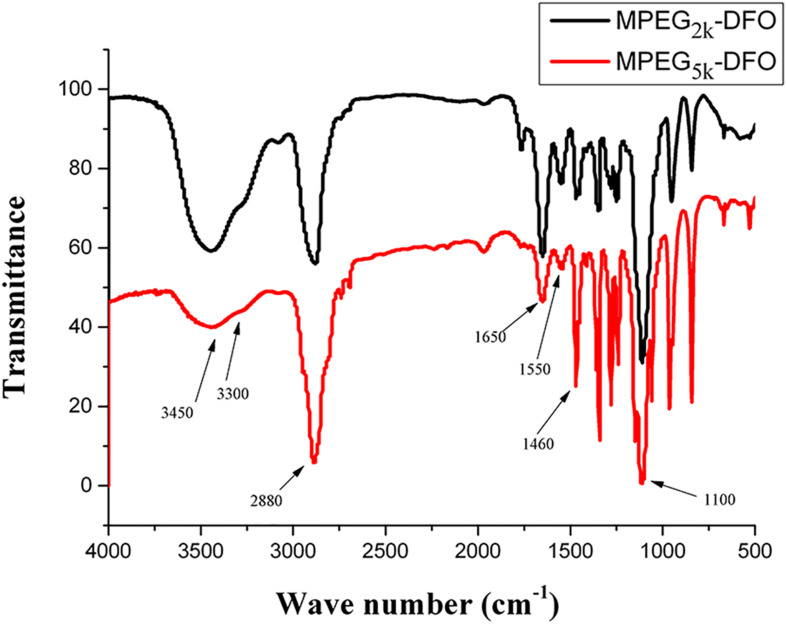
FTIR spectra of PEGylated DFO.

PEGylated DFO was synthesized by EDC chemistry, where the amino group in DFO was coupled to the carboxylate group in carboxylated mPEG. The FTIR spectra of the obtained products were shown in Figure 2. Both products exhibited similar spectra: the broadband around 3,450 cm^–1^ was assigned to the N-H and O-H stretching vibrations; the band at 3,300 cm^–1^ was attributed to the N-H stretching vibrations, and the band at 2,880 cm^–1^ was assigned to the symmetric stretching vibrations of the CH_2_; the band at 1,650 and 1,550 cm^–1^ were assigned to amide I and II, respectively; the band at 1,460 cm^–1^ was attributed to the bending vibration of the CH_2_, and the band at 1,100 cm^–1^ was assigned to stretching vibrations of the C-O-C. These results indicated that amide bonds were formed in the products.

**FIGURE 3:**
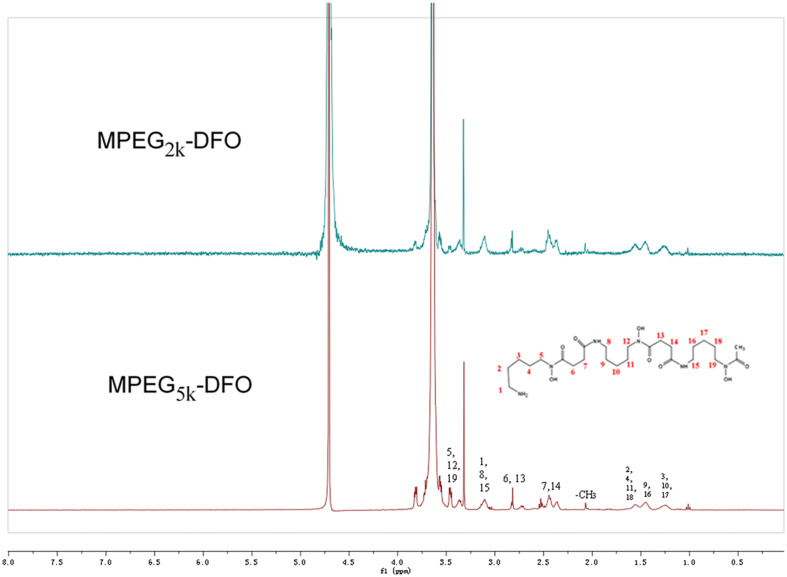
^1^HNMR spectra of PEGylated DFO.

To further confirm the chemical structure of the synthesized products, ^1^HNMR measurements were carried out in this study. Both products exhibited similar ^1^HNMR spectra (Figure 3). The signals at 3.6 and 3.3 ppm were assigned to the protons of CH_3_O- and CH_2_- in the mPEG, respectively. The signal at 3.4 ppm was assigned to H-5, H-12, and H-9 in DFO. The signal at 3.1 ppm was assigned to H-1, H-8, and H-15 in DFO. The signal at 2.8 ppm was assigned to H-6 and H-13 in DFO. The signals at approximately 2.5 ppm were assigned to H-7 and H-14 in DFO. The signal at 2.0 was assigned to protons of CH_3_- in DFO. The signal at 1.5 ppm was assigned to H-2, H-4, H-11, and H-18 in DFO. The signal at 1.4 ppm was assigned to H-9 and H-16 in DFO, and the signal at 1.2 ppm was assigned to H-3, H-10, and H-17 in DFO. Together, these results suggested that the DFO had been successfully PEGylated.

### Chelation Ability of PEGylated DFO

**FIGURE 4:**
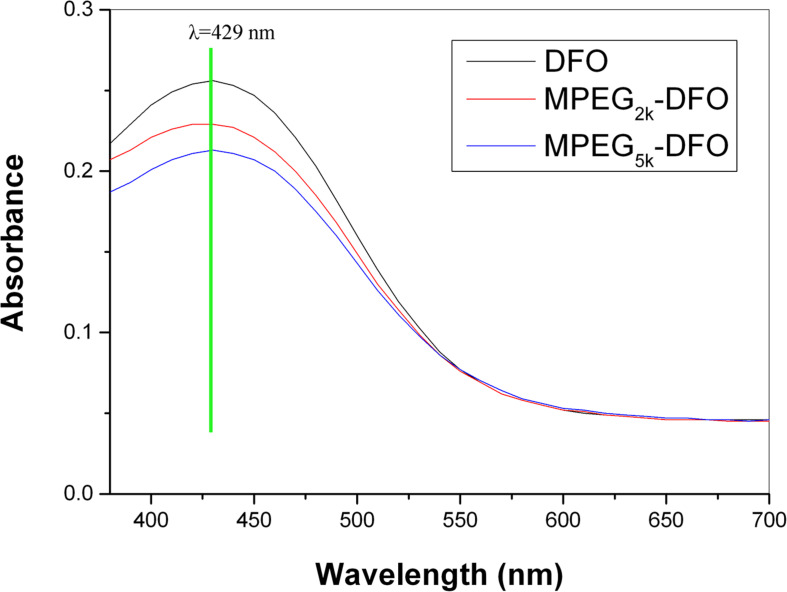
Full-length UV-Vis scanning of PEGylated DFO.

When DFO chelates with iron, the DFO converts to the iron-saturated complex, ferriox-amine, which has a characteristic absorption at 429 nm (Hallaway et al., 1989). To check if the PEGylated DFO could preserve the chelation ability of the DFO, full-length UV–vis scanning was performed. Both products exhibited similar characteristic absorption to that of DFO with the maximum at 429 nm (Figure 4), indicating that the iron-chelating ability of DFO was not compromised after PEGylation. The amounts of DFO in the products were determined according to the standard curve. The results of the analysis showed 0.89 and 0.83 of DFO molecules coupled per mPEG chain for mPEG_2__k_-DFO, and mPEG_5__k_-DFO, respectively.

### Stability Assay

**FIGURE 5:**
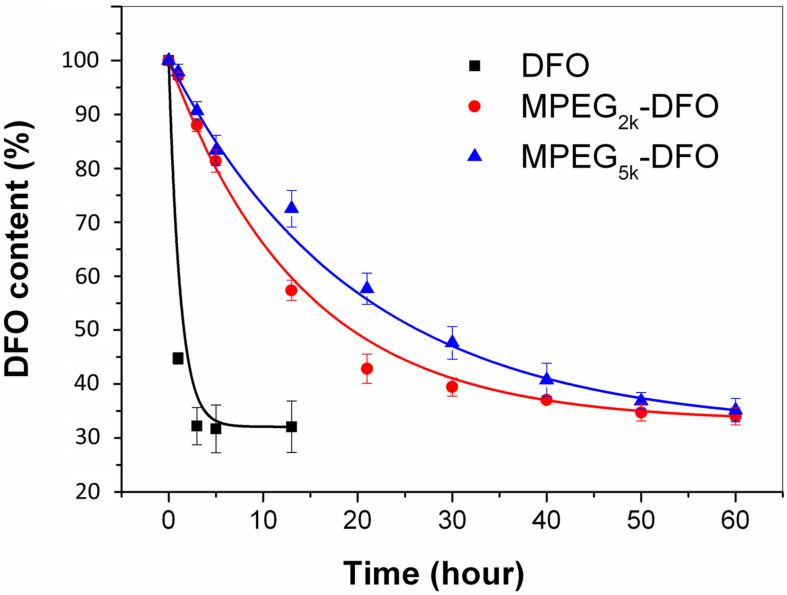
Metabolism assay for the PEGylated DFO.

One of the most common shortcomings of DFO is that it is unstable in plasma, resulting from metabolization by α_2_-globulins and loss of its ability to bind iron, resulting in a short half-life. The metabolism assays of both the PEGylated DFO and DFO showed that DFO was metabolized rapidly, and its content decreased by more than 50% in 1 h (Figure 5). In contrast, PEGylated DFO exhibited much longer stability compared with DFO. The half-lives calculated from the fitting curves showed that the DFO had a short half-life (0.8 h), whereas the mPEG_2__k_-DFO (18.2 h) and mPEG_5__k_-DFO (27.8 h) had significantly longer half-lives, respectively (*P* < 0.05). This was more than 20-fold longer than that of the DFO and resulted from the hydrophilic characteristics of the PEG molecular chains. This characteristic effectively resisted the adsorption of the proteins (such as enzymes) and decreased the degradation of the coupled drugs. Here, we also observed that the half-life of the PEGylated DFO was proportional to the molecular weight of the PEG. This finding was consistent with previous reports suggesting that longer PEG chains offer better protection from enzyme degradation (Wang et al., 2010).

### Cytotoxicity

**FIGURE 6:**
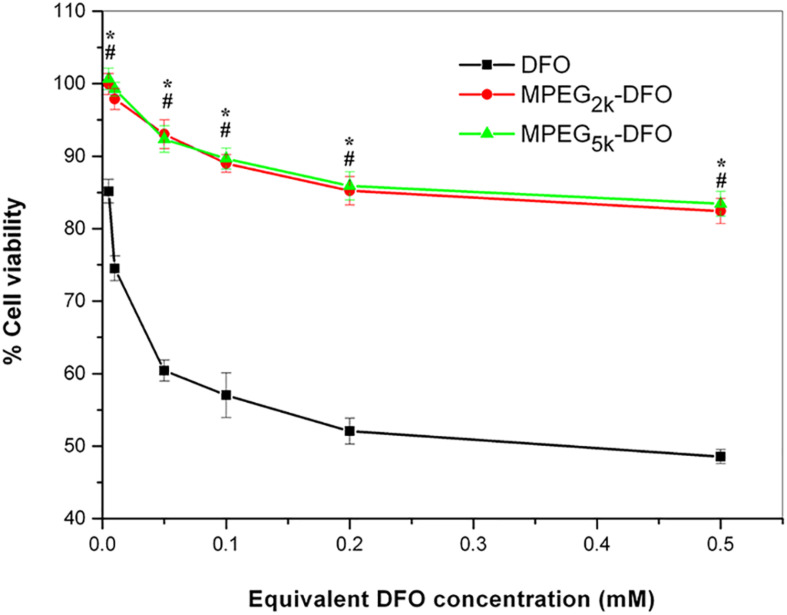
Cell viability of HUVECs in the presence of the PEGylated DFO. “*”: DFO vs. MPEG_2__*K*_-DFO, *P* < 0.05; “#”: DFO vs. MPEG_5__*K*_-DFO, *P* < 0.05.

Cytotoxicity is a significant shortcoming of DFO that acts in a dose- and time-dependent manner. To investigate whether PEGylated DFO can improve the cytotoxicity of DFO, the cell viability was assessed after treatment with a series of concentrations of the PEGylated DFO or DFO and assayed using the MTT method. The cell viability measurements of the PEGylated DFO groups was significantly higher compared with the DFO groups for every corresponding equivalent DFO concentration (*P* < 0.05, Figure 6). Specifically, the cell viability of the DFO group was lower than 75% when the concentration of the DFO was above 0.05 mM, and it decreased to 48.6% at 0.5 mM DFO. In contrast, the cell viability measurements of the PEGylated DFO groups were approximately 85% or greater at all equivalent DFO concentrations. Therefore, it can be concluded that PEGylation decreased the cytotoxicity of DFO.

### Hemocompatibility

**FIGURE 7:**
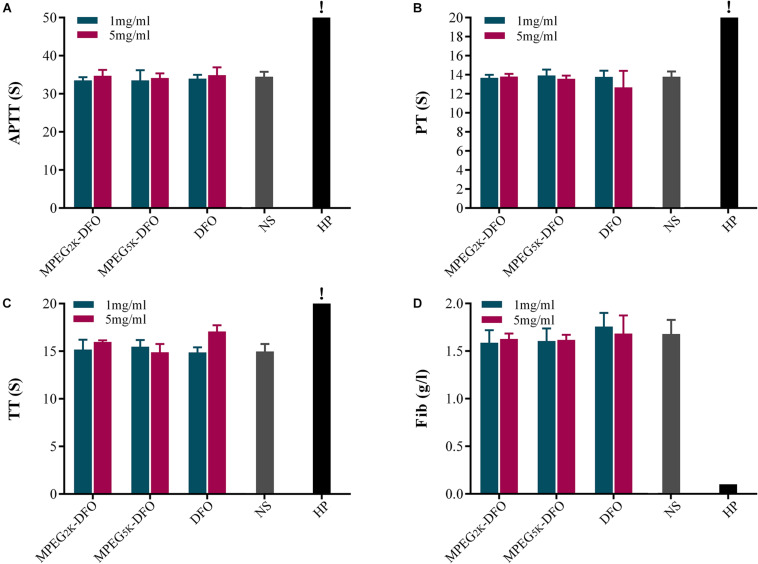
Effect of PEGylated DFO on the coagulation system. **(A)** APTT, **(B)** PT, **(C)** TT, and **(D)** Fib were tested by an automated coagulation analyzer. The values were too high to be measured (APTT > 245 s, TT > 169 s). HP, heparin. !: The values are too high that cannot be determined by the analyzer.

Four indexes, including APTT, PT, TT, and Fib, were calculated to determine the effect of PEGylated DFO on the coagulation system (Siedlecki, 2018). The results showed that, for APTT, both concentrations (1 and 5 mg/ml) of PEGylated DFO were not significantly different from the saline group (Figure 7A). Additionally, all the PT values were in the normal range (Figure 7B). For TT, except that the higher concentration (5 mg/ml) of DFO slightly prolonged the TT, both PEGylated DFO concentrations are similar to the saline group (Figure 7C). Besides, the concentrations of Fib in all groups were similar to that in the saline group without significant difference (Figure 7D).

The balance between coagulation and anticoagulation is crucial to the human body. In general, interactions between the contact phase of the coagulation system and biomaterials leads to an induction of the intrinsic coagulation, and thrombosis may be an outcome of this process (Ekdahl et al., 2011). APTT, PT, TT, and Fib are involved in intrinsic, extrinsic, and common pathways of blood coagulation. Our results showed that there was no significant difference between each index in the PEGylated DFO group and that in the saline group, which indicated that PEGylated DFO had no significant effect on the coagulation system.

**FIGURE 8:**
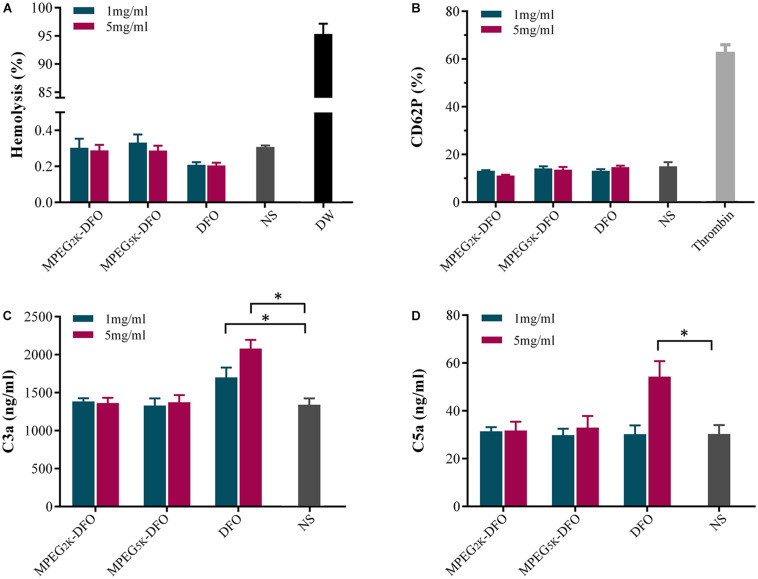
Effect of PEGylated DFO on hemolysis, platelet activation, and complement activation. **(A)** The percent of hemolysis. **(B)** Platelet activation. **(C)** The content of C3a. **(D)** The content of C5a. *p < 0.05.

The hemolysis assay is used to evaluate the hemocompatibility of biomaterials, and it is performed by detecting hemoglobin released from lysed RBCs (Guo X. et al., 2018). Materials can be classified as hemolytic if they induce over 5% hemolysis, slightly hemolytic (2–3%), and non-hemolytic (below 2%) (Weber et al., 2018). The percent of hemolysis induced by the mPEG_2__k_-DFO, mPEG_5__k_-DFO, and DFO groups fluctuated between 0.2 and 0.4%. Additionally, there was no statistical difference when compared with the saline group, which means that PEGylated DFO has a low risk of inducing hemolysis (Figure 8A).

To investigate the effects of PEGylated DFO on platelet activation, flow cytometry was used to detect the level of the fluorescent platelet activation marker against CD62P (Sun et al., 2019). The results indicated that there was no significant difference in platelet activation between the PEGylated DFO and saline groups, suggesting that PEGylated DFO barely influenced the platelet activation (Figure 8B).

As a primary component of the innate immune system, complement activation is regarded as an essential factor of hemocompatibility (Ekdahl et al., 2011). When the biocompatibility of biomaterials is poor, the main pathway activated is the alternative pathway, which is associated with the creation of the anaphylatoxins C3a and C5a (Mödinger et al., 2018). In our study, the concentrations of C3a and C5a in plasma exposed to PEGylated DFO were measured as indicators of complement activation. mPEG_2__k_-DFO and mPEG_5__k_-DFO showed similar complement activation when compared with the saline group. In contrast, the activation of C3a and C5a induced by 5mg/ml DFO was significantly higher compared with the saline group (Figures 8C,D, C3a: 2079.76 ± 114.25 vs. 1342.41 ± 84.51; C5a: 54.28 ± 6.48 vs. 30.30 ± 3.74, *P* < 0.05, respectively), which suggests that the PEGylated DFO induces little to no complement activation in this condition.

### Iron Staining

**FIGURE 9:**
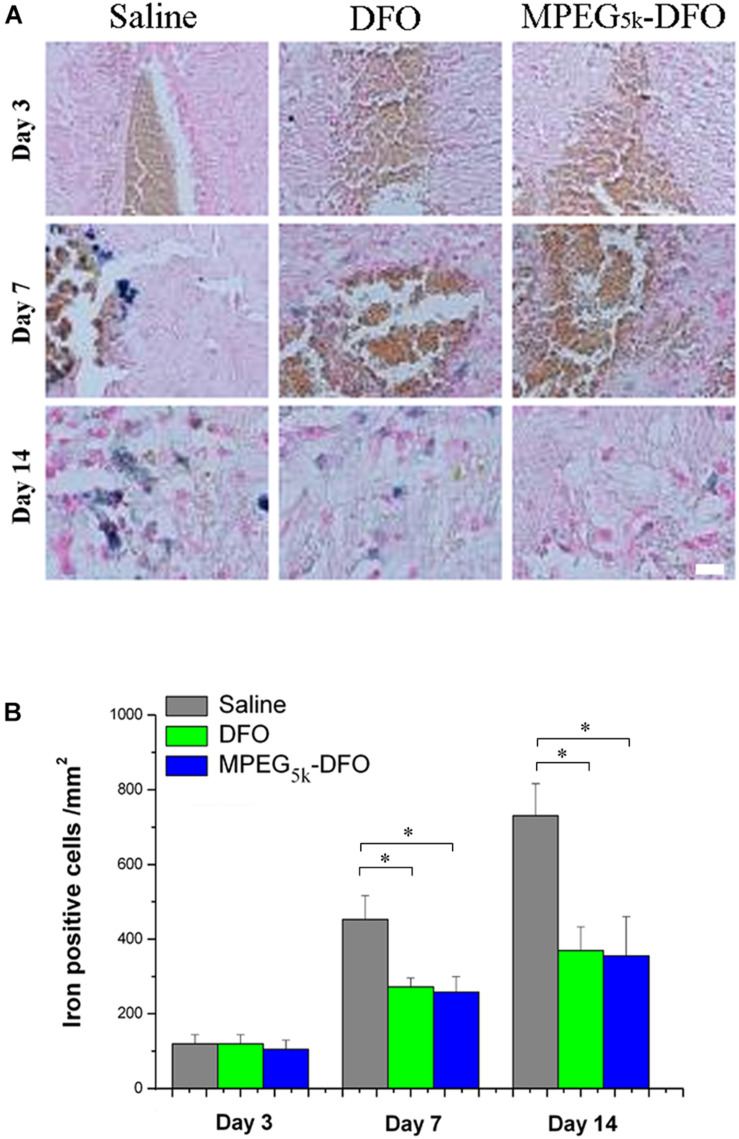
Iron deposition was evaluated by iron staining. **(A)** Representative images for iron staining. **(B)** Quantification of the iron-positive cells. ^∗^*p* < 0.05, scale bar, 20 μm.

An increasing number of studies have found that hemoglobin-derived iron-overload plays a significant role in secondary injury after stroke (Garton et al., 2016). Thus, it is critical to decreasing accumulated iron after stroke. Iron staining showed that the positive cells increased gradually from day 3 to 14 in all groups (Figures 9A,B). Specifically, there was no difference among the three groups on day 3. However, on days 7 and 14, mPEG_5__k_-DFO and DFO treatment significantly reduced the number of iron-positive cells compared with the saline group (7D: DFO/mPEG_5__k_-DFO vs. Saline: 257.08 ± 24.76/259.25 ± 39.59 vs. 456.40 ± 65.31; 14D: DFO/mPEG_5__k_-DFO vs. Saline: 375.51 ± 63.84/359.18 ± 106.86 vs. 740.14 ± 87.20. *P* < 0.05, respectively). This was consistent with a previous study that demonstrated that DFO was capable of reducing the iron accumulation after stroke (Guo et al., 2019). In our study, we showed that the mPEG_5__*K*_-DFO group contained only one-tenth of the iron load compared with the DFO group, but it exhibited a similar iron reduction capacity. This suggested that the same therapeutic effect could be achieved with the administration of fewer DFO doses, which seems to be a feasible approach to reduce DFO clinical medication.

### FJC Staining

**FIGURE 10:**
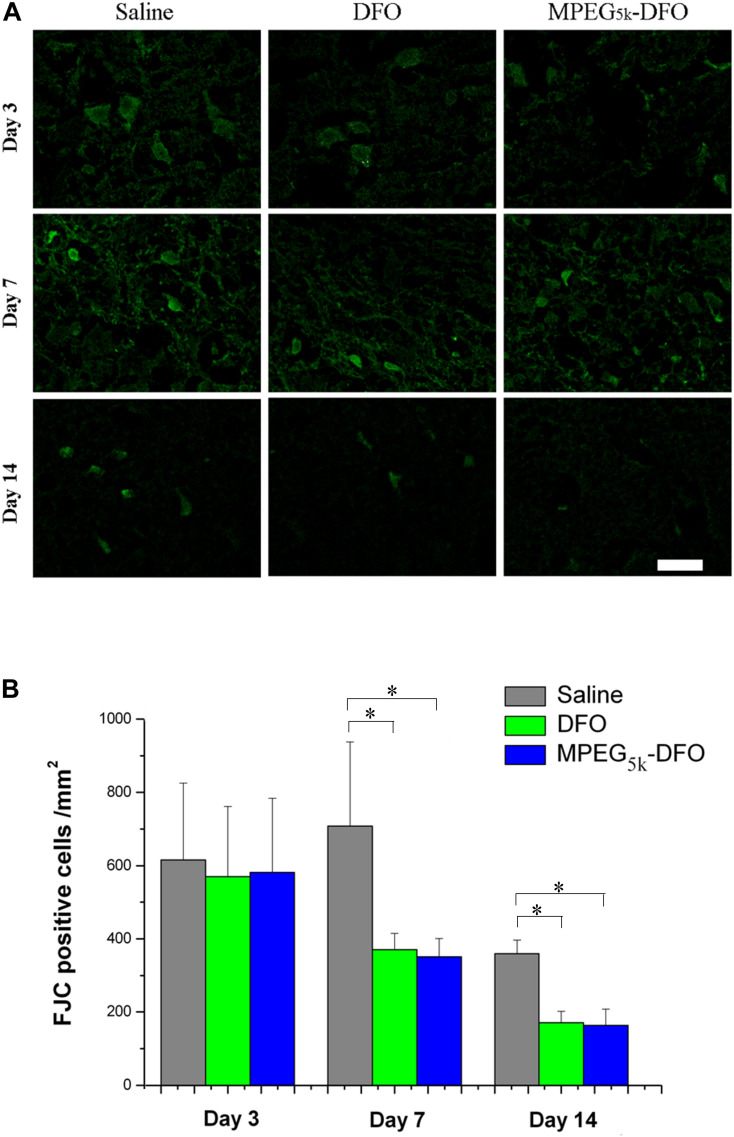
The detection of the degenerated neurons. **(A)** Representative images for FJC staining of degenerated neurons. **(B)** Quantification of the FJC positive cells. ^∗^*p* < 0.05, scale bar, 20 μm.

To detect neuronal damage after a stroke, the degeneration of the neurons was assayed using FJC staining. On day 3, there was no significant difference in FJC-positive cells among the three groups. However, on days 7 and 14, the FJC-positive cells in both mPEG_5__k_-DFO and DFO groups were significantly decreased compared with the saline group (Figure 10, 7D: DFO/mPEG_5__k_-DFO vs. Saline: 374.17 ± 46.07/356.02 ± 48.18 vs. 718.31 ± 232.47; 14D: DFO/mPEG_5__k_-DFO vs. Saline: 173.12 ± 30.02/164.40 ± 45.03 vs. 364.39 ± 35.60. *P* < 0.05, respectively). In addition, there was no significant difference between the mPEG_5__k_-DFO and DFO groups on days 7 and 14.

Following a stroke, neurons may be damaged by necrosis, apoptosis, autophagic cell death, and ferroptosis (Shao et al., 2019), which seriously affect the density of neurons around the lesions and cause serious nerve function injury (Chen et al., 2019; Xu et al., 2020). Our results showed that mPEG_5__*K*_-DFO exhibited a similar therapeutic effect (in reducing the degeneration of neurons) compared with DFO, which might result from their iron-chelating effect (Guo et al., 2019).

### Histopathology of Organs

Microscopic examination and qualitative analysis of the heart, liver, spleen, lung, and kidney showed no gross or microscopic differences between the normal, Saline, DFO, and mPEG_5__k_-DFO groups on Supplementary Figure S1, which imply that both DFO and mPEG_5__k_-DFO have no hazardous effects on the organs of heart, liver, spleen, lung, and kidney on rats.

### Neurological Behavior

**FIGURE 11:**
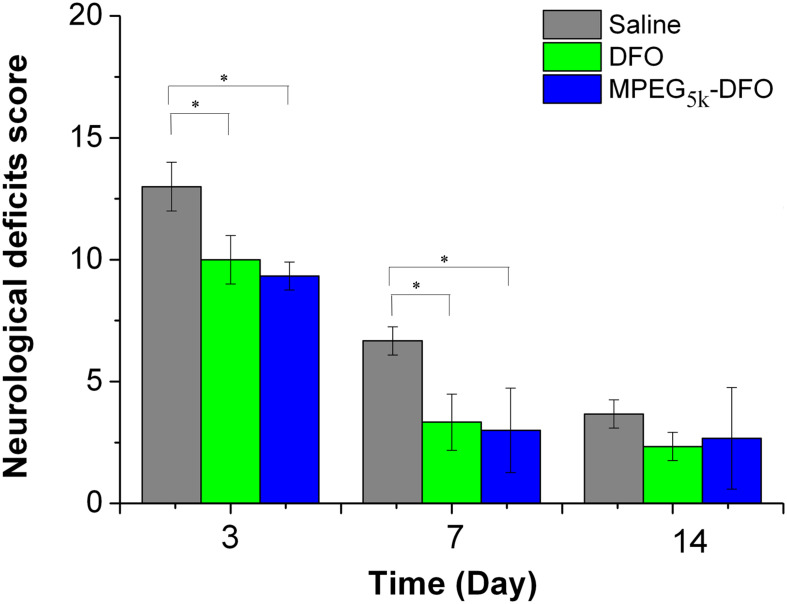
Quantification of the neurological deficit testing. ^∗^*p* < 0.05.

To evaluate the therapeutic effect of PEGylated DFO on nerve function defects following a stroke, neurological deficit scoring at different time points was performed. The neurological deficit scores for all three groups decreased gradually over time, indicating recovery of function (Figure 11). Moreover, on days 3 and 7, both mPEG_5__*K*_-DFO and DFO groups showed a significant decrease in scores compared with the saline group (3D: DFO/mPEG_5__k_-DFO vs. Saline: 10.08 ± 0.99/9.36 ± 0.54 vs. 13.03 ± 1.00; 7D: DFO/mPEG_5__k_-DFO vs. Saline: 3.32 ± 1.12/2.97 ± 1.71 vs. 6.68 ± 0.58. *P* < 0.05, respectively). This suggested that there was a functional recovery in both the mPEG_5__*K*_-DFO and DFO groups compared with the saline group. On day 14, there was no significant difference between all three groups. In general, the nerve function recovery of the rodent model post-stroke is time-dependent, or it may be spontaneous recovery (Manaenko et al., 2011). This is why, by day 14, the neurological behavior of all the groups was restored to a reasonable level as previously reported (Karki et al., 2009; Yang et al., 2011). The density of the neurons around the lesion is closely related to the recovery of nerve function injury (Chen et al., 2019; Xu et al., 2020). The FJC staining showed that both mPEG_5__*K*_-DFO and DFO treatment could reduce the number of degenerated neurons, which may explain, in part, why both the mPEG_5__*K*_-DFO and DFO groups exhibited better recovery of function compared with the saline group.

## Conclusion

In conclusion, PEGylated DFO was successfully synthesized by EDC chemistry, and the structure was characterized. A chelation assay confirmed that PEGylated DFO exhibited a similar characteristic absorption to that of DFO with the maximum at 429 nm. The metabolism assay showed that the PEGylated DFO had a half-life more than 20-fold greater than that of the DFO. With regards to compatibility, the PEGylated DFO had lower cytotoxicity than that of the DFO and no significant effect on blood components. In the iron-overload stroke model in rats, the PEGylated DFO decreased the iron accumulation, neuronal degeneration, and promoted recovery of function. Therefore, the synthesized PEGylated DFO in this work has a strong potential to be used in applications for treating iron-overload conditions in the clinic.

## Data Availability Statement

All datasets presented in this study are included in the article/Supplementary Material.

## Ethics Statement

The studies involving human participants were reviewed and approved by the Ethical Committee of Institute of Blood Transfusion, Chinese Academy of Medical Sciences and Peking Union Medical College. The patients/participants provided their written informed consent to participate in this study. The animal study was reviewed and approved by the Animal Ethical Committee of Sichuan University.

## Author Contributions

JX wrote the manuscript and the discussion of the results. TS and RZ performed part of the experiments. CY was involved in the discussion of the results. MT was responsible for conceptualizing, performing the experiments, the discussion of the results, and revising the manuscript. All authors contributed to the article approved the submitted version.

## Conflict of Interest

The authors declare that the research was conducted in the absence of any commercial or financial relationships that could be construed as a potential conflict of interest.
