# Supplementary material for: PEGylation of Deferoxamine for Improving the Stability, Cytotoxicity, and Iron-Overload in an Experimental Stroke Model in Rats
Source: Front Bioeng Biotechnol. 2020 Sep 25;8:592294. doi: 10.3389/fbioe.2020.592294 (PMC7546414; doi:10.3389/fbioe.2020.592294)
Supplement: Supplementary file 1 [file Image_1.pdf]

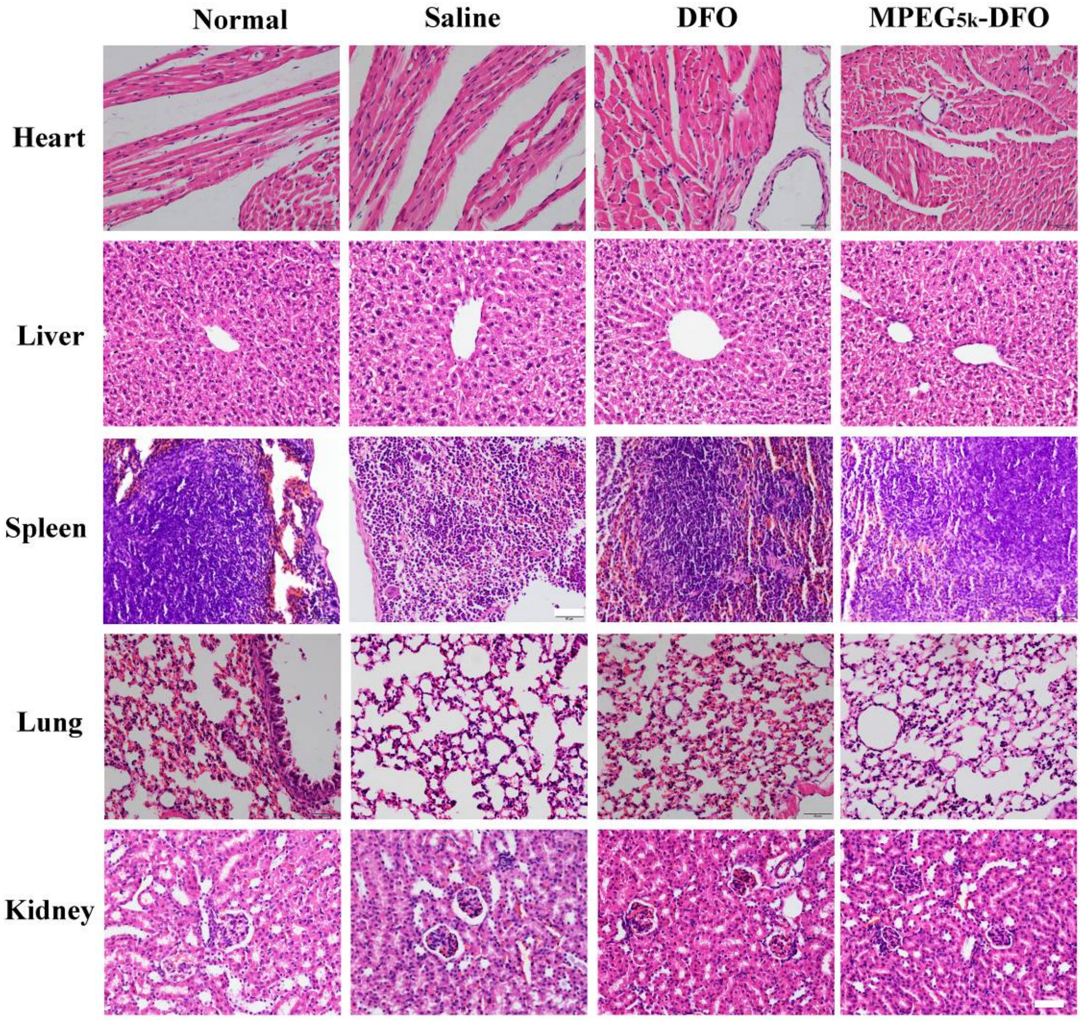

Figure S1. Representative H.E. staining photomicrographs of the heart, liver, spleen, lung, and kidney of normal, Saline, DFO, and $MPEG_{5k}$-DFO groups. Scale bar: 50 μm.
